# Inflammatory markers and long term hematotoxicity of holmium-166-radioembolization in liver-dominant metastatic neuroendocrine tumors after initial peptide receptor radionuclide therapy

**DOI:** 10.1186/s13550-022-00880-4

**Published:** 2022-02-02

**Authors:** Sander C. Ebbers, Tessa Brabander, Margot E. T. Tesselaar, Johannes Hofland, Manon N. G. J. A. Braat, Frank J. Wessels, Maarten W. Barentsz, Marnix G. E. H. Lam, Arthur J. A. T. Braat

**Affiliations:** 1 Department of Radiology and Nuclear Medicine, University Medical Center Utrecht, Heidelberglaan 100, 3584 CX Utrecht, The Netherlands; 2 Department of Radiology and Nuclear Medicine, Erasmus Medical Center, Doctor Molewaterplein 40, 3015 GD Rotterdam, The Netherlands; 3 Department of Medical Oncology, Netherlands Cancer Institute, Plesmanlaan 121, 1066 CX Amsterdam, The Netherlands; 4 Department of Internal Medicine, Erasmus Medical Center, Doctor Molewaterplein 40, 3015 GD Rotterdam, The Netherlands

**Keywords:** PRRT, NET, Neuroendocrine tumor, Neuroendocrine neoplasm, Lutetium-177-dotatate, Radioembolization, Holmium-166, Hematologic toxicity, NLR, TLR, Inflammatory markers

## Abstract

**Purpose:**

In patients with neuroendocrine tumor liver metastases, additional tumor reduction can be achieved by sequential treatment with [^166^Ho]-radioembolization after peptide receptor radionuclide therapy (PRRT). The aim of this study was to analyze hematotoxicity profiles, (i.e. lymphocyte and neutrophile toxicity) and the prognostic value of neutrophil-to-lymphocyte ratio (NLR) and thrombocyte-to-lymphocyte ratio (TLR).

**Methods:**

All patients included in the prospective HEPAR PLuS study were included in this study. Blood testing was performed at baseline (before radioembolization) and at regular intervals during 1-year follow-up. Radiological response was assessed at 3, 6, 9, and 12 months according to RECIST 1.1. Logistic regression was used to analyze the prognostic value of NLR and TLR on response.

**Results:**

Thirty-one patients were included in the toxicity analysis; thirty were included in the response analysis. Three weeks after radioembolization, a significant decrease in lymphocyte count (mean change − 0.26 × 10^9^/L) was observed. Ten patients (32.2%) experienced grade 3–4 lymphocyte toxicity. This normalized at 6 weeks and 3 months after treatment, while after 6 months a significant increase in lymphocyte count was observed. An increase in NLR and TLR at 3 weeks, compared to baseline, significantly predicted response at 3 months (AUC = 0.841 and AUC = 0.839, respectively) and at 6 months (AUC = 0.779 and AUC = 0.765). No significant relation with survival was found.

**Conclusions:**

Toxicity after sequential treatment with PRRT and [^166^Ho]-radioembolization is limited and temporary, while significant additional benefit can be expected. Change in NLR and TLR at 3-weeks follow-up may be valuable early predictors of response.

*Trial registration* ClinicalTrials.gov, NCT02067988. Registered 20 February 2014, https://clinicaltrials.gov/ct2/show/record/NCT02067988.

**Supplementary Information:**

The online version contains supplementary material available at 10.1186/s13550-022-00880-4.

## Key points

- **Question**

Are inflammatory markers a relevant predictor of response after [^166^Ho]-radioembolization in neuroendocrine tumor patients?

- **Pertinent findings**

In a cohort study, following 31 patients who underwent [^166^Ho]-radioembolization shortly after PRRT, a temporary increase in neutrophil-to-lymphocyte ratio (NLR) and thrombocyte-to-lymphocyte ratio (TLR) three weeks after treatment was found to be indicative of objective response at three months.

- **Implications for patient care**

NLR and TLR may be of use in both identifying early response and providing insight in the biological process of tumor response after [^166^Ho]-radioembolization in neuroendocrine tumor patients.

## Introduction

Peptide receptor radionuclide therapy (PRRT) with [^177^Lu]Lu-DOTA-TATE is standard of care in patients with somatostatin receptor (SSTR) positive grade 1 or 2 gastroenteropancreatic neuroendocrine tumors (NET). It is indicated following progression after initial treatment with somatostatin analogs (SSA). However, large liver metastases (i.e. > 3 cm) or a high tumor burden (i.e. > 25%) indicate worse overall and disease-specific survival after PRRT [1, 2]. The additional benefit of radioembolization/selective internal radiotherapy (SIRT) to boost the effect of PRRT was recently studied in the HEPAR PLuS study, as well as the hepatic toxicity of combining both treatments [3]. After [^166^Ho]-radioembolization of one or both liver lobes, objective response was achieved in 43% of the patients, and toxicity was limited. However, within a 6-month follow-up period, grade 3 lymphopenia was observed in 23% of patients. This is a phenomenon that is known to occur after both PRRT and radioembolization, and is thought to be caused by the immune response initiated after radiation therapy of the tumors, direct irradiation of white blood cells, and targeting of B-lymphocytes by overexpression of SST_2_-receptors [4, 5]. Furthermore, some studies showed a relationship between baseline neutrophil-to-lymphocyte ratio (NLR) or thrombocyte-to-lymphocyte ratio (TLR) and recurrence or survival after surgery and chemoembolization in NET [6–8]. A high NLR or TLR at baseline was associated with a worse prognosis after different treatment strategies in various types of tumor, among which breast, colorectal, gastric, oesophageal, pancreatic, lung, renal, bladder, ovarian, hepatocellular and cholangiocarcinoma [9]. As markers of the systemic inflammatory response in cancer patients, NLR and TLR are thought to be of prognostic value. Therefore, the NLR and TLR were also proposed as prognostic factors after radioembolization [10].

It is not known whether the combined treatment of PRRT and [^166^Ho]-radioembolization has an additional effect on post-treatment lymphopenia and hematological toxicity in general. In this study, the 1-year hematotoxicity was assessed after combined therapy consisting of PRRT and [^166^Ho]-radioembolization.

## Materials and methods

### Patients

Data was analyzed from the prospective HEPAR PLuS study, which was conducted at the University Medical Center Utrecht, The Netherlands (Clinical Trials identifier: NCT02067988) [3, 11]. Patients with a well differentiated NET (grade 1–2) were treated with additional [^166^Ho]-radioembolization after receiving four cycles of PRRT with [^177^Lu]Lu-DOTA-TATE. The full protocol and primary endpoints were published previously [3, 12]. In short, patients were eligible for inclusion in de HEPAR PLuS study if they were ≥ 18 years old; had Eastern Cooperative Oncology Group (ECOG) performance score ≤ 2; had at least three measurable liver metastases according to Response Evaluation Criteria In Solid Tumors version 1.1 (RECIST 1.1); had treatment with [^166^Ho]-radioembolization within 20 weeks after the final cycle of PRRT. Patients were excluded if laboratory toxicity grade 3 or higher according to the Common Terminology Criteria for Adverse Events (CTCAE 4.03) was present, except for lymphopenia and γ-glutamyltransferase, for which all toxicity was accepted. Written informed consent was obtained from all participants. The local medical ethics committee approved the study and the study was conducted according to the declaration of Helsinki.

### Study procedures

Blood samples were taken at multiple time points: at screening (i.e. 2–4 weeks before [^166^Ho]-radioembolization), on the day of treatment, 3 and 6 weeks, and 3, 6, 9, and 12 months after treatment with [^166^Ho]-radioembolization. If available, baseline laboratory tests before PRRT were collected as well (pre-PRRT). All patients were planned to receive a mean target volume absorbed dose of 60 Gy. To determine response after [^166^Ho]-radioembolization, contrast-enhanced computed tomography of the chest (single-phase) and abdomen (multi-phase) was performed at baseline screening and at 3, 6, 9, and 12 months after treatment.

### Outcomes

Toxicity was classified according to the CTCAE 4.03 and was assessed in each patient at each time point independently. Toxicity profiles at screening (i.e. prior to [^166^Ho]-radioembolization) were defined as baseline, and only parameters that showed changes compared to baseline according to CTCAE 4.03 were reported. The NLR and TLR were calculated by dividing the absolute neutrophil count or absolute thrombocyte count by the absolute lymphocyte count, respectively. Pre-existing hematotoxicity after PRRT was quantified at baseline (i.e. prior to [^166^Ho]-radioembolization), with the aim to assess its effect on post-[Ho^166^]-radioembolization hematotoxicity.

Two radiologists, who were blinded for patient characteristics, independently assessed response. The objective response at 3, 6, 9, and 12 months after [^166^Ho]-radioembolization was assessed using RECIST 1.1. If disagreement between the two radiologists occurred, the mean of both sums of the diameter of the target lesions was calculated and the response was re-classified. Patients discontinued follow-up in the study if they showed either clinical or radiological progression of disease and/or were referred for additional treatment.

### Statistical analysis

Categorical variables, including the response after treatment using RECIST 1.1 criteria, were compared by means of Chi-square or Fisher’s exact test, depending on group size. Differences in continuous variables were tested using *t*-tests. When analyzing predictors for lymphocytopenia, linear mixed models were used, to incorporate the correlation within the longitudinal data of laboratory tests. Tested variables were gender, age, tumor grade, hepatic tumor burden (defined as the fraction of the liver volume occupied by tumor tissue, segmented on CE-CT), ECOG score, extrahepatic disease (excluding lymph nodes), and total liver dose (Gy), because these factors were considered potential clinically relevant prognostic factors. Individual testing of potential factors in univariate analysis was performed, as well as multivariate analysis. Logistic regression analysis was performed to test the predictive value of baseline and change in NLR and TLR at 3 and 6 weeks post-treatment on the occurrence of objective response according to RECIST 1.1 at 3, 6, 9, or 12 months. Objective response was defined as partial or complete response. In order to improve comparability of the developed models, response assessment at 6, 9, and 12 months was inflated to the same amount of cases of which response was available at 3 months, by counting cases that were lost to follow-up as showing progressive disease. Backward step model reduction was used to reduce the model with the full set of predictors to a reduced model containing only significantly contributing predictors. From the resulting models, ROC curves were constructed and if multiple predictor variables were included, optimism-corrected area-under-the-curve (AUC) values were calculated by bootstrapping the dataset. Odds ratios (OR) with the corresponding confidence intervals (CI) were calculated. The total follow-up time of patients was used for calculating the overall survival (OS), censoring those patients that were lost to follow-up due to other reasons than death. Progression-free-survival (PFS) was determined for each patient, censoring patients showing enduring response at the time of analysis. A possible relation between NLR and TLR and OS or PFS was tested using Cox Proportional Hazards Regression analysis, from which hazard ratios (HR) and CIs were calculated.

Statistical analysis was performed in R (version 4.0.1). All statistical tests were performed two-sided. A *p *value of < 0.05 was considered significant.

## Results

In total, 31 patients were included in the analysis (Table 1). A total of two out of 31 patients (6%) died before follow-up was completed. Follow-up was prematurely ended in 3/31 patients (9%) because of progressive disease, which required initiation of a new therapy. Follow-up was completed in all other patients (26/31 patients; 84%). Median time between the last cycle of PRRT and treatment with [^166^Ho]-radioembolization was 12.7 weeks (IQR [9.7; 15.8]). Any unresolved hematotoxicity after PRRT (CTCAE grade ≥ 1) was found in 27 patients (87%). At baseline (i.e. after PRRT, prior to [^166^Ho]-radioembolization), grade 1–2 lymphopenia was observed in 11 patients (35.5%), grade 3 lymphopenia in 7 (22.6%), grade 1 leucopenia in 11 (35.5%), and grade 1–2 anemia in 17 (55%, Table 2). Grade 1 renal toxicity was present in 3 patients (9.7%) and bilirubin elevation was absent in all patients. When only considering newly observed toxicity after PRRT (i.e. compared to pre-PRRT results), lower CTCAE grades were observed at baseline, prior to radioembolization (Table 3).

**Table 1:** Baseline characteristics of included patients in the HEPAR-PLuS trial

	N (%)
Number of patients	31
Age—Median (IQR)	65.1 (57.6–70.2)
Gender	
Male	23 (74.2%)
Female	8 (25.8%)
Origin of tumor	
Pancreas	10 (32.3%)
Small intestine	8 (25.8%)
Colorectal	4 (12.9%)
Lung	3 (9.7%)
Unknown primary	6 (19.4%)
WHO Grade	
1	12 (38.7%)
2	19 (61.3%)
Weeks since final cycle PRRT—Median (IQR)	12.7 (9.7–15.8)
ECOG status	
0	17 (54.8%)
1	13 (41.9%)
2	1 (3.2%)
Total liver dose in Gy—Median (IQR)^a^	46.4 (41.2–55.1)
Tumor burden in %—Median (IQR)	6.9% (3.1–22.5%)
Extrahepatic disease	
Absent	11 (35.5%)
Lymph nodes	5 (16.1%)
Visceral	6 (19.4%)
Lymph nodes and visceral	9 (29.0%)
Baseline NLR—Median (IQR)	4.3 (3.6–5.7)
Baseline TLR—Median (IQR)	238 (176–308)

*IQR* interquartile range

^a^Absorbed dose as calculated based on the total liver volume and the total infused activity. This does not equal 60 Gy in all patients due to not all patients receiving a total liver treatment

**Table 2:** Raw baseline toxicity prior to PRRT and at screening (i.e. prior to [^166^Ho]-radioembolization)

	CTCAE grade					
	Pre-PRRT			Baseline		
	1–2	3	4	1–2	3	4
Thrombocyte	3/30			12/31		
Neutrophil	2/26			1/31		
Lymphocyte		1/27		11/31	7/31	
Leukocyte	3/30			11/31		
Hemoglobin	13/30			17/31		

Values are *n/available* observed patients with the designated toxicity grade

**Table 3:** All new observed hematological and biochemical toxicity after [^166^Ho]-radioembolization during 1 year of follow-up

	CTCAE grade								
	Baseline (Pre-[^166^Ho]-radioembolization)^a^			0–12 months post-[^166^Ho]-radioembolization			End of follow-up (1 year)^b^		
	1–2	3	4	1–2	3	4	1–2	3	4
Thrombocyte	8/30			9/31			2/26		
Neutrophil				5/31			2/26		
Lymphocyte	10/27	5/27		7/31	9/31	1/31	1/26	1/26	
Leukocyte	10/30			11/31			5/26		
Hemoglobin	6/30			8/31	1/31		4/26		

Values are *n/available* observed patients with the maximum observed toxicity grade during the designated period of follow-up. CTCAE grades at pre-[^166^Ho]-radioembolization were counted when the grade was higher than at pre-PRRT, CTCAE grades in post-[^166^Ho]-radioembolization follow-up were counted when the grade was higher than at baseline

^a^Several values not available due to missing data at pre-PRRT

^b^Five patients did not reach the 12-month follow-up

**Fig. 1:**
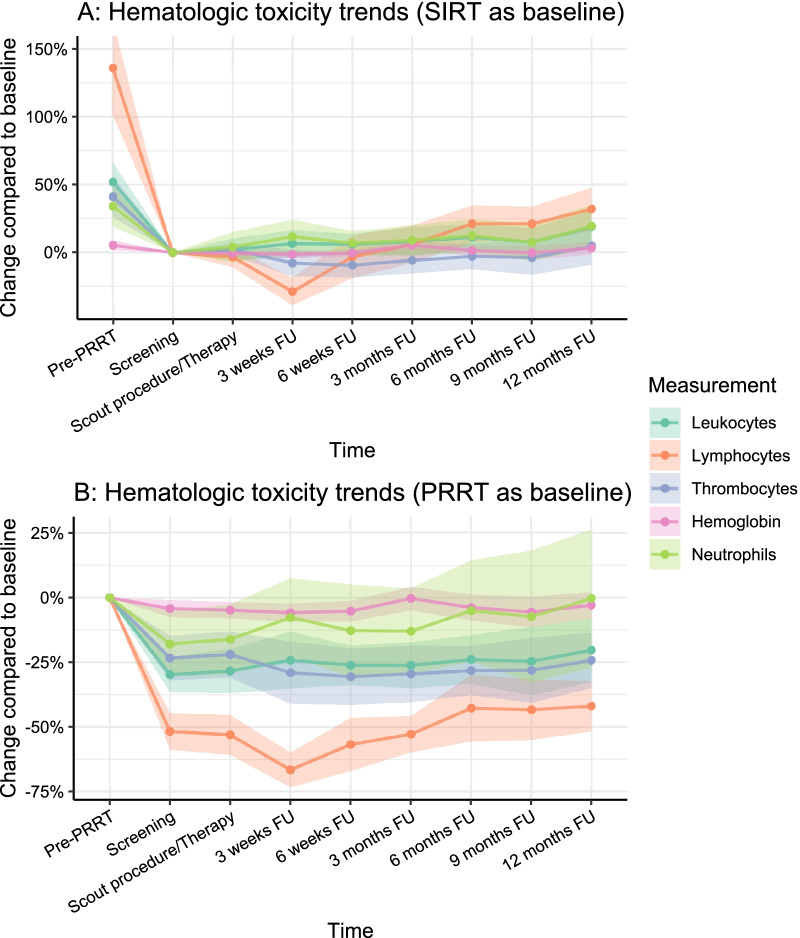
Laboratory test trends indicating hematologic toxicity. Points indicate median change compared to baseline. *FU* follow-up

Most hematotoxicity was caused by PRRT, but some additional toxicity after [^166^Ho]-radioembolization was observed (Fig. 1). After 3 weeks of follow-up, a significant further reduction in average lymphocyte levels was observed. Compared to baseline, this change was more pronounced than any of the other hematologic tests. Grade 3–4 lymphopenia occurred in ten patients (32.2%) and grade 1–2 lymphopenia in seven patients (22.6%; Table 3). Lymphocyte counts rapidly increased in the following weeks and months. There was a significant decrease at 3-weeks follow-up (− 0.26 × 10^9^/L; *p* < 0.001) compared to baseline, and an increase at six (+ 0.14 × 10^9^/L), nine (+ 0.13 × 10^9^/L), and twelve (+ 0.19 × 10^9^/L) months (*p* = 0.023, *p* = 0.039 and *p* = 0.003 respectively). The increase in lymphocyte counts at 6, 9, and 12 months indicated further recovery from pre-existent hematotoxicity after PRRT. No patient- or disease specific factors were associated with lymphocytic toxicity at any point in time during follow-up (Additional file 1: Table S1).

Hemoglobin, leukocyte, and thrombocyte specific hematotoxicity was limited (Table 3). In terms of CTCAE grading, one patient (3.2%) suffered from grade 3 anemia, eight patients (25.8%) had grade 1–2 anemia, eleven patients (35.5%) had grade 1–2 leukopenia, and nine patients (29.1%) had grade 1–2 thrombopenia. For all hematologic laboratory values, a recovery trend was observed in the second half-year of follow-up.

Response assessment (according to RECIST 1.1) was available in 30 patients at 3-months follow-up, 29 patients at 6-months follow-up, 28 at 9-months follow-up, and 25 at 12-months follow-up. After 3, 6, 9, and 12 months, partial response was observed in 43%, 47%, 37%, and 37%, stable disease in 43%, 37%, 30% and 30%, and progressive disease in 13%, 17%, 33% and 33% respectively. One patient died before any response assessment could be performed.

**Fig. 2:**
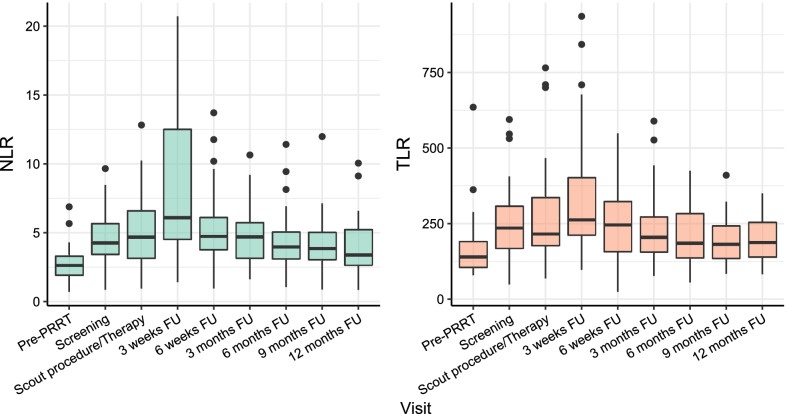
Trends in absolute NLR and TLR after [^166^Ho]-radioembolization, with a significant peak 3 weeks after treatment. *FU* follow-up

**Fig. 3:**
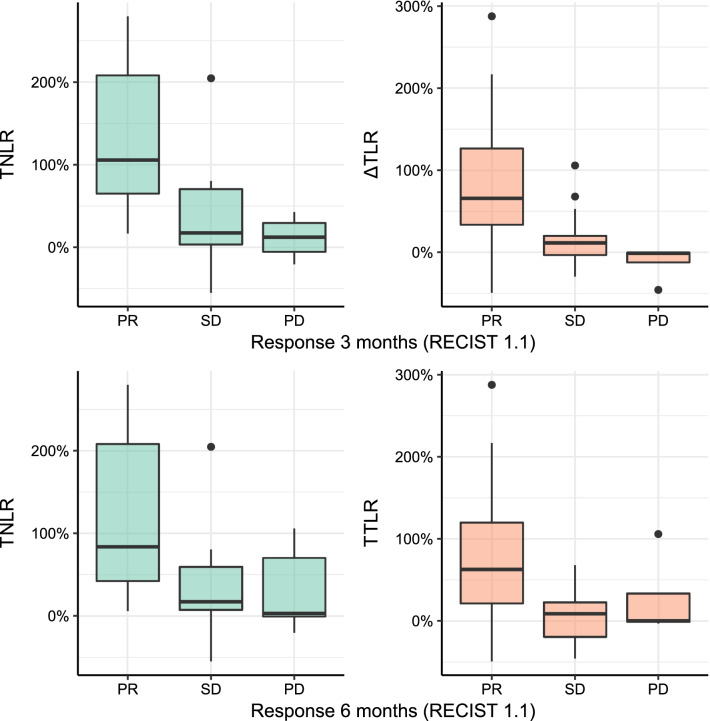
Differences in relative change in NLR and TLR at 3-weeks follow-up after [^166^Ho]-radioembolization plotted against response according to RECIST 1.1 at 3 months (top-left and top-right) and 6 months (bottom-left and bottom-right)

**Fig. 4:**
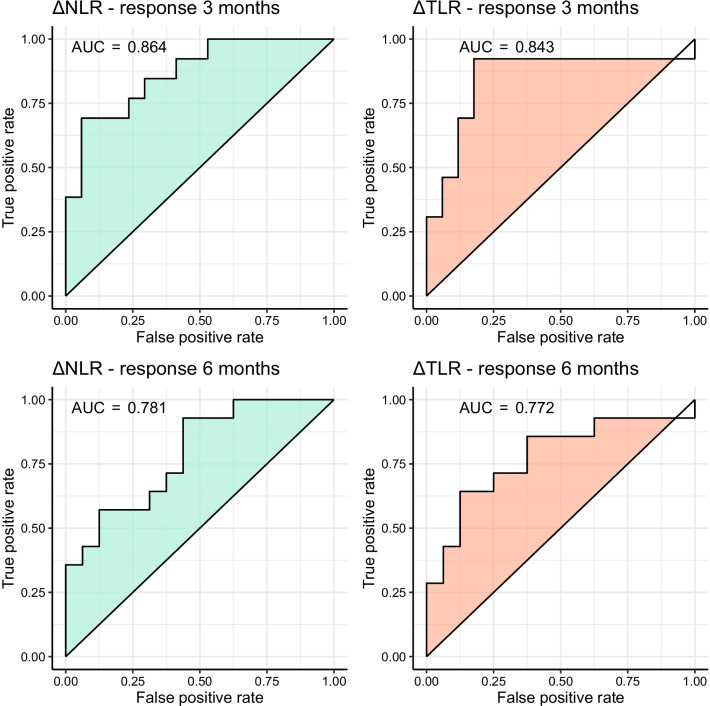
ROC curves of relative change in NLR and TLR from baseline at 3-weeks follow-up in predicting objective response at 3 months (top row) and 6 months (bottom row), according to RECIST 1.1. *AUC* area under the curve

**Fig. 5:**
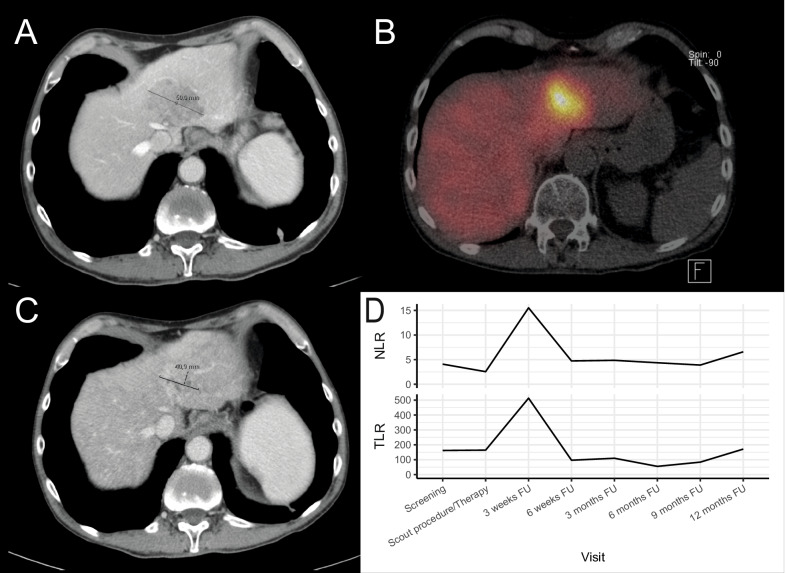
A male patient with hepatic neuroendocrine tumor metastases, with the largest lesion measuring 60 mm at screening (**A**), was treated with whole-liver [^166^Ho]-radioembolization (**B**). After 3 months partial response was observed, with the largest lesion measuring 41 mm (**C**). A clear peak in NLR and TLR can be observed at 3 weeks follow-up (**D**). *FU* follow-up

In 22 patients, a temporary increase in NLR or TLR (of whom 13 had an increase > 50%) was observed at 3-weeks follow-up, which recovered during further follow-up (Fig. 2). Median baseline NLR was 4.3, median baseline TLR was 238 (Table 1). Correlation between NLR and TLR within patients was moderate at baseline (*r* = 0.57, *p* < 0.001), 6-weeks follow-up (*r* = 0.67, *p* < 0.001), and 12-months follow-up (*r* = 0.52, *p* = 0.001). Correlation between NLR and TLR was strong at 3-weeks follow-up (*r* = 0.84, *p* < 0.001), as well as the correlation between change in NLR and TLR after 3 weeks (*r* = 0.77, *p* < 0.001). NLR or TLR did not differ between patients with grade 1 or grade 2 NET at any point in time (*p* > 0.05). The increase in NLR and TLR can be mostly attributed to a mean decrease in lymphocytes of 28.8% (95% CI [18.6; 39.1]), and in a lesser degree to an increase in neutrophils (mean increase 11.7%, 95% CI [− 0.8; 24.2]) or decrease in thrombocytes (mean decrease 7.7%, 95% CI [− 2.0; 17.5]). Baseline NLR and TLR did not predict response at three (OR_NLR_ = 1.05, 95% CI [0.72; 1.54] and OR_TLR_ = 1.00, 95% CI [0.99; 1.00]) or 6 months FU (OR_NLR_ = 0.93, 95% CI [0.64; 1.36] and OR_TLR_ = 1.00, 95% CI [0.99; 1.01]). The relative change in both NLR and TLR at 3-weeks follow-up was significantly associated with response at 3 months based on RECIST 1.1, meaning that an increase in NLR or TLR could predict response to treatment (OR per 100% increase NLR = 7.06, 95% CI 1.47–34.03, *p* = 0.001 and OR per 100% increase TLR = 14.33, 95% CI 1.66–123.4, *p* = 0.001; Fig. 3). The relative change in NLR and TLR at 3-weeks follow-up was also significantly associated with response at 6 months (OR per 100% increase NLR = 4.08, 95% CI 1.20–13.94, *p* = 0.001 and OR per 100% increase TLR = 7.59, 95% CI 1.23–46.96, *p* = 0.001). The change in NLR was also significantly associated with response at 9 and 12 months (OR per 100% increase NLR = 2.78, 9%% CI 1.05–7.34), while the change in TLR was not significantly associated with response at 9 or 12 months (*p* = 0.08). At 6-weeks follow-up, the change in NLR and TLR could not predict response at 3 months (*p* = 0.402 and *p* = 0.318 respectively). The ROC curve of using change in NLR or TLR in the current dataset yielded an AUC of 0.864 and 0.843 for predicting response at 3 months, and an AUC of 0.781 and 0.772 for predicting response at 6 months (Fig. 4). Adding both variables in the model did not improve the bootstrap-corrected AUC (AUC = 0.821). Using several cut-offs, sensitivity and specificity were calculated (Additional file 2: Table S2). An example of a patient showing a marked peak in NLR and TLR at 3 weeks while having partial response on imaging at 3 months is shown in Fig. 5.

**Fig. 6:**
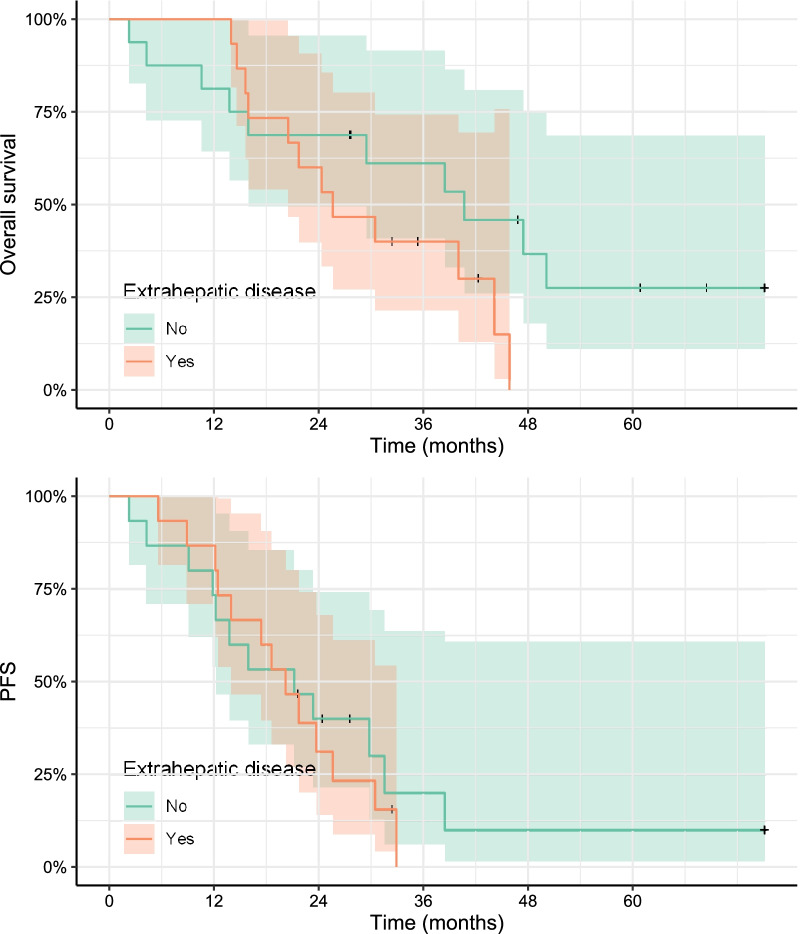
Difference in overall survival and progression-free survival in patients with or without extrahepatic disease (excluding lymph node involvement)

Median OS in the entire study population (*n* = 31) was 30.5 months after [^166^Ho]-radioembolization (mean 35.4, 95% CI [21.7; 47.4]), and 40.8 months after start of PRRT (mean 44.4, 95% CI [31.2; 56.3]). Median PFS was 21.2 months (mean 28.4, 95% CI [14.0; 31.5]) after [^166^Ho]-radioembolization, and 30.1 months after PRRT (mean 37.9, 95% CI [24.7; 40.8]). Nine patients were alive at the time of analysis, and five patients did not show progression in the time they were followed-up. No significant relation could be found between change in NLR or TLR and OS, with a HR per 100% increase in NLR of 0.8262 (95% CI 0.49–1.39, *p* = 0.474) and a HR for TLR of 0.97 (95% CI 0.49–1.92, *p* = 0.94), nor for PFS (HR_NLR_ = 0.98, 95% CI [0.65; 1.62]; HR_TLR_ = 1.03, 95% CI [0.58; 1.82]). Presence of extrahepatic disease did not influence median OS (41 vs 26 months, *p* = 0.14) or median PFS significantly (21 vs 20 months, *p* = 0.619; Fig. 6).

## Discussion

After sequential PRRT and [^166^Ho]-radioembolization, additional short-term lymphopenia was observed in 55% of the treated patients with well-differentiated (grade 1–2) NET, 32% had grade 3–4 lymphopenia. After 12 months, additional lymphopenia was present in only two patients. Other hematologic parameters (i.e. thrombocytes, neutrophils, leukocytes and hemoglobin) were mainly limited to grade 1–2 toxicity. Additionally, trends indicated that after a radiation boost to the liver using [^166^Ho]-radioembolization, patients recovered from additional radioembolization-related toxicity, and partially from pre-existent PRRT-related hematologic toxicity within 1 year of follow-up. In the current study, no baseline factors could be identified that correlated with an increased risk of hematologic toxicity. Most notably, the NLR and TLR at 3-weeks follow-up after [^166^Ho]-radioembolization seem to be predictors of response at 3 months after treatment according to RECIST 1.1. Specifically, an increase of NLR or TLR compared to baseline improved the chance of objective response in our population. This observation was independent of pre-existing lymphopenia, indicating that lymphopenia is not a contra-indication for [^166^Ho]-radioembolization.

Multiple studies assessed the toxicity profiles after yttrium-90 [^90^Y]-radioembolization monotherapy in patients with hepatic NET metastases. In a study by Zuckerman et al*.*, using glass microspheres (TheraSphere™, Boston Scientific), grade 3 lymphopenia occurred in 14/59 patients (23.7%), similar to the number of patients with grade 3 lymphopenia in the current study [13]. Thrombopenia was mainly limited to grade 1 or 2 toxicity, further confirming our findings. In another study, performed by Tomozawa et al*.*, 52 patients with hepatic NET metastases were followed 1 year after [^90^Y]-radioembolization using resin microspheres (SIR-Spheres; Sirtex Medical Ltd), of whom 11 were followed 4 years. Within 1 year, hematologic toxicity occurred rather infrequently, with no grade 3 hematologic toxicity, and grade 1–2 anemia, grade 1–2 thrombopenia, and grade 1–2 leukopenia in only a limited number of patients. However, the WHO grade was not reported and only late (6–12-months) follow-up was presented, while in the current study hematologic toxicity occurred after 3 weeks. In a previously published study by Braat et al*.*, 244 patients with well-differentiated (grade 1–3) NET treated with resin [^90^Y]-radioembolization were included in a retrospective international multicenter study [14]. Toxicity during follow-up was very comparable with this study, with grade 1–2 lymphopenia occurring in 52% of patients, grade 1–2 thrombopenia in 17%, and grade 1–2 anemia or leukopenia in less than 8%. Lymphopenia was the most frequent cause for grade 3–4 toxicity. However, hematologic toxicity was only analyzed up to 3 months after treatment. The current study showed similar toxicity profiles compared to previously published studies on [^166^Ho]-radioembolization [15, 16]. Lymphocyte levels showed the most significant toxicity. Overall, given the available toxicity profiles after radioembolization, there seems to be only limited added hematotoxicity from radioembolization when given sequentially after PRRT.

In earlier publications, the value of NLR and TLR was studied in different fields of medicine and for different types of tumors [6, 8]. It is well established, that baseline NLR can predict the OS, PFS and disease-free survival for many cancers, including colorectal cancer, breast cancer, hepatocellular carcinoma, gastric cancer, esophageal cancer and NET [17–22]. A high NLR is thought to be an indicator of systemic inflammatory response, and can be used as an index for severity of disease in cancer patients [23]. From the acknowledged hallmarks of cancer, inflammatory response can be seen as both a cause for as well as a result from tumor growth [24–26]. Thus, a high NLR is thought to reflect a more severe disease phenotype. In most studies, for a wide variety of treatments, both systemic (such as chemotherapy) and locoregional (such as radiotherapy), NLR is used as a baseline index for the systemic inflammatory status of patients, their capability to induce reduction in tumor growth, and therefore response to treatment and survival [6–8, 10]. In the current study, an increase in NLR or TLR shortly after treatment appeared to be indicative of response at 3 and 6 months, which contrasts earlier publications, even though the currently presented relation is between change in NLR and TLR and response, rather than baseline NLR and TLR. For example, Estrade et al*.* found that in HCC patients, lymphopenia 3 months after radioembolization, and therefore an increased NLR, was associated with poor survival (14.3 months vs 23.4 months) [27]. The difference might be due to the effect of radioembolization on short-term systemic availability of lymphocytes, granulocytes and thrombocytes. Therefore, the observed increase in NLR and TLR must be seen more as a treatment effect, than as a disease effect. It is also noteworthy that the short-term NLR and TLR trends (i.e. change within one month after radioembolization) have not been studied before. Furthermore, there may be certain unknown confounders that explain the observed relationship. The pre-[^166^Ho]-radioembolization NLR and lymphocyte counts seems to be in accordance with ranges found earlier in NET patients, so a bias in patient selection seems unlikely [22, 28].

Another marker recently studied in NET is the inflammation-based index (IBI), based on c-reactive protein and albumin levels [29]. It was demonstrated by Black et al*.* to show prognostic value in hepatocellular patients, and was proposed as a selection tool for PRRT in NET patients [30–32]. In the study, an increased IBI was found to be a significant prognosticator for decreased overall and progression-free survival, while NLR and TLR at baseline were not found to be significant prognosticators. This approach may be analogous to measuring NLR and TLR at baseline, as both markers represent the inflammatory status of the patient. However, the role of the inflammatory status of the patient in patient selection is not yet fully understood. As both PRRT and radioembolization may be beneficial even in patients with a high inflammatory status, it is unfeasible to withhold therapy from these patients. Therefore, this study proposes these inflammatory markers to be used during follow-up as well, as these parameters may be predictive of response.

For [^90^Y]-radioembolization, it was shown that an increase in baseline NLR or TLR was indicative of a worse prognosis in patients with primary or secondary liver malignancies [10]. In the study by D’emic et al*.*, patients with hepatocellular carcinoma, colorectal cancer, breast cancer, bile duct cancer or NET, OS and PFS were significantly worse in patients with higher NLR and TLR at 20 days post-treatment in univariate analysis. In multivariate analysis, a post-treatment increase in TLR was most significantly associated with worse OS and PFS [10]. However, the study population was very heterogeneous, with a wide variety of tumor types included, among which only 8 (6.8%) patients with NET. Contrary to the study by D’emic, the current study focused on response in relation to the relative change in NLR and TLR after [Ho^166^]-radioembolization, in patients with NET. A temporary increase in NLR at 3 weeks post-treatment was associated with objective response at 3, 6, 9, and 12 months, which may seem contrary to previous findings. However, we could not find a relation with overall survival. It is important to note that our study is the first study to focus solely on the relation between NLR and TLR and response in neuroendocrine tumor patients. This is essentially different from studies focusing on the prognostic value of baseline NLR and TLR, as the change in NLR and TLR after treatment may reflect the effect of the treatment on the disease, rather than baseline NLR and TLR, which reflect the disease status of the patient prior to treatment. This is also essentially different from NLR and TLR several months post-treatment, which may potentially be obscured by disease progression and subsequently increased systemic inflammation. Although the prognostic value of NLR (and TLR) has been evaluated in multiple studies, it is rarely used in clinical practice.

There are some limitations in this study. First, the sample size for prognostic studies in general should preferably be larger. Unfortunately, multivariate prognostic models are difficult to build in small samples, as frequently encountered in NET studies. Second, there was some missing data, as not all patients completed follow-up. However, toxicity data up to and including the 3 weeks follow-up visit and response after three months was available in all patients. Third, our study consists of both grade 1 and grade 2 NET, which tend to have a very different course of disease over the years. Because of this heterogeneity, survival analysis is less accurate. Finally, all patients in the study were treated with a mean target liver volume absorbed dose of 60 Gy, which was calculated using the MIRD model which assumes the injected activity to be distributed evenly throughout the healthy liver tissue and tumor tissue. Toxicity in patients treated with [^166^Ho]-radioembolization can be further reduced by calculating the therapeutic activity using the so-called partition or multicompartment model [33].

Patients who are initially treated with PRRT can be safely referred for additional [^166^Ho]-radioembolization, as a boost in treatment of liver metastases [3]. In clinical practice, a significant treatment boost can safely be pursued in patients with bulky NET liver disease. Based on this study, measurement of NLR and TLR at baseline and during short-term follow-up visits may provide early information on response to treatment. In the future, prospective studies on the benefit of monitoring inflammatory markers on overall and progression-free survival should be conducted, because much is still unclear. Especially in patients with NET, predictive and prognostic markers may be difficult to find due to the heterogeneous nature of the tumor.

In conclusion, no clinically significant or permanent additional hematologic toxicity was observed after sequential treatment with PRRT and [^166^Ho]-radioembolization, while inflammatory markers such as NLR or TLR may provide early information on treatment response.

## Supplementary Information

**Additional file 1.** Mixed model analysis of lymphocyte levels at 3 weeks follow-up.

**Additional file 2.** Test characteristics of NLR and TLR.

## Data Availability

The data that support the findings of this study are available upon reasonably request via contacting the corresponding author.

## Abbreviations

PRRT: Peptide receptor radionuclide therapy
SSTR: Somatostatin receptor
NET: Neuroendocrine tumor
SIRT: Selective internal radiotherapy
NLR: Neutrophil-to-lymphocyte ratio
TLR: Thrombocyte-to-lymphocyte ratio
