# Supplementary material for: Inflammatory markers and long term hematotoxicity of holmium-166-radioembolization in liver-dominant metastatic neuroendocrine tumors after initial peptide receptor radionuclide therapy
Source: EJNMMI Res. 2022 Feb 2;12:7. doi: 10.1186/s13550-022-00880-4 (PMC8811020; doi:10.1186/s13550-022-00880-4)
Supplement: Supplementary file 1 — Additional file 1. Mixed model analysis of lymphocyte levels at 3 weeks follow-up. [file 13550_2022_880_MOESM1_ESM.docx]

## Supplemental table 1

Relation between various patient characteristics and lymphocyte levels at 3 weeks follow-up, using mixed linear models, testing individual variables (univariate) and all variables in one model (multivariate)

* extrahepatic disease indicates presence of any tumor depositions measurable according to RECIST 1.1, excluding lymph nodes

| Variable | | Univariate analysis | p-value | Multivariate analysis | p-value |
| --- | --- | --- | --- | --- | --- |
| Gender | |  |  |  |  |
|  | Female | ref |  | ref |  |
|  | Male | 0.0492 | 0.723 | 0.028 | 0.865 |
| Age | | -0.005 | 0.446 | -0.010 | 0.210 |
| Tumor grade | |  |  |  |  |
|  | 1 | ref |  | ref |  |
|  | 2 | 0.0915 | 0.461 | -0.172 | 0.230 |
| Tumor burden | | 0.0529 | 0.886 | -0.297 | 0.497 |
| ECOG | |  |  |  |  |
|  | 0 | ref |  | Ref |  |
|  | 1 | -0.131 | 0.296 | 0.167 | 0.286 |
|  | 2 | -0.0959 | 0.783 | 0.439 | 0.271 |
| Total liver dose (Gy) | | -0.008 | 0.159 | -0.009 | 0.196 |
| Time since PRRT | | 0.0161 | 0.181 | 0.0001 | 0.995 |
| Extrahepatic disease* | | 0.091 | 0.453 | 0.062 | 0.663 |
