# Supplementary material for: Inflammatory markers and long term hematotoxicity of holmium-166-radioembolization in liver-dominant metastatic neuroendocrine tumors after initial peptide receptor radionuclide therapy
Source: EJNMMI Res. 2022 Feb 2;12:7. doi: 10.1186/s13550-022-00880-4 (PMC8811020; doi:10.1186/s13550-022-00880-4)
Supplement: Supplementary file 2 — Additional file 2. Test characteristics of NLR and TLR. [file 13550_2022_880_MOESM2_ESM.docx]

## Supplemental table 2

Test characteristics of change in NLR and TLR in predicting response at three months according to RECIST 1.1

| NLR change - cutoff | | Sensitivity | Specificity |
| --- | --- | --- | --- |
|  | 17% | 0.9231 | 0.4706 |
|  | 40% | 0.8462 | 0.5882 |
|  | 45% | 0.7692 | 0.7059 |
|  | 71% | 0.6923 | 0.8235 |
|  | 83% | 0.6154 | 0.9412 |
| TLR change - cutoff | |  |  |
|  | 0% | 0.9231 | 0.4706 |
|  | 13% | 0.9231 | 0.6471 |
|  | 23% | 0.9231 | 0.8235 |
|  | 41% | 0.6923 | 0.8235 |
|  | 62% | 0.5385 | 0.8824 |
